# Unusual conservative treatment of a complicated pacemaker pocket infection: a case report

**DOI:** 10.1186/s13256-019-1987-x

**Published:** 2019-03-03

**Authors:** Wanqiu Kang, Xiaoming Chen, Zicheng Li, Aidong Zhang, Jingwen Liu, Liqiong Yu, Yingzhen Wen

**Affiliations:** 1 The First Clinical Medical College of Jinan University, Guangzhou, 510630 China; 2 Department of Cardiology, Guangzhou Overseas Chinese Hospital, The First Affiliated Hospital of Jinan University, Guangzhou, 510630 China

**Keywords:** Complicated pacemaker pocket infection, Conservative treatment, Partial removal, Microbes, Antimicrobial therapy, Temporary pacing, Reimplantation

## Abstract

**Background:**

For patients with complicated generator pocket infection, expert consensuses universally advocate complete device and leads removal followed by delayed replacement on the contralateral side. We cured our patient by partial generator removal and reimplantation of sterilized pulse generator on the ipsilateral side. We also performed a literature review about incomplete removal therapy for the management of cardiac implantable electronic device infection.

**Case presentation:**

An 86-year-old Chinese Han man was diagnosed as having third-degree atrioventricular block and received a permanent double-chamber pacemaker in his left prepectoral area 15 years ago. Nine years later, the entire system was removed because of confirmed infection, and a new device was reimplanted in the contralateral area. He developed skin necrosis around the pacemaker pocket after 1 year, and his generator was renewed without leads extraction at another medical center. He was subsequently admitted several times for surgical tissue debridement at another institution due to extended skin necrosis. At the time of the new admission, he had severe infection, heart failure, and hypoalbuminemia. He was diagnosed as having complicated pacemaker pocket infection. Our preferred treatment strategy was for complete removal of both the generator and transvenous pacing leads, and we intended to implant an epicardial pacemaker in our patient if necessary. However, he rejected the treatment strategy and firmly refused to replace his generator. We had to attempt a novel pacemaker-preserving strategy considering our patient’s severe comorbidities. Finally, we cured him by partial generator removal and reimplantation of the sterilized pulse generator on the ipsilateral side. There was no sign of wound dehiscence or infection during a 6-month follow-up.

**Conclusions:**

We would posit that partial removal of infected generators combined with conservative treatment may be a proper treatment of complicated generator pocket infection, especially for those who are susceptible to cardiac complications. Reimplantation of a sterilized pulse generator on the ipsilateral side may be an option if patients reject a new device and contralateral vascular condition is not really suitable. Opting for such treatment should be at the consideration of the primary care physician based on the condition of the patient.

## Background

An expansion in the clinical indications for implantation of cardiovascular implantable electronic devices (CIEDs), such as bradycardia, tachyarrhythmia, and heart failure, has led to a significant increase in the use of CIEDs over the past several decades [1, 2]. Concurrently, CIED infection (CIEDI) has become increasingly prevalent [3]. Sandoe *et al.* defined complicated pacemaker pocket infection as pacemaker pocket infection with evidence of lead involvement, systemic symptoms of infection, or positive blood cultures [4]. For patients with complicated pacemaker pocket infection, expert consensuses universally advocate complete device and leads removal followed by delayed replacement on the contralateral side [4–7]. Unfortunately, some patients may not be candidates for device removal due to multiple comorbidities, limited life expectancy, or personal preference, which leads to reassessment of the optimal management strategies for these infections. We report a case of a patient with complicated pacemaker pocket infection who was cured by partial generator removal, reimplantation of the sterilized pulse generator on the ipsilateral side, debridement, and antimicrobial therapy. Few studies in the literature have reported such conservative treatment.

## Case presentation

An 86-year-old Chinese Han man, with a known history of hypertension, heart failure, and chronic kidney disease, was diagnosed as having third-degree atrioventricular block and received a permanent double-chamber pacemaker in the left prepectoral area 15 years ago. Nine years later, the entire system (generator and leads) was removed because of confirmed infection, and a new device was reimplanted in the contralateral area. Unfortunately, he developed skin necrosis around the pacemaker pocket after 1 year and the generator was renewed without leads extraction at another medical center. After this procedure, a focal area at the mid portion of the wound failed to fully heal. He was subsequently admitted several times due to extended skin necrosis with massive purulent secretion and cellulitis around the incision site. His primary physician used multiple courses of antibiotics, local wound care, and debridement. This conservative management was continued for 5 years at another institution. There was ongoing pressure necrosis of the overlying skin which led to the gradual extrusion of his leads.

No social, environmental, family, or employment histories were related to his illness. He was born in China and has been living in Guangzhou for nearly 60 years. There is no hereditary disease in his family. He has a son who is in good health. He was an engineer before he retired 26 years ago. The following orally administered medications were given regularly to control his hypertension, heart failure, and chronic kidney disease in other hospitalizations: benazepril (10 mg once daily), niaoduqing (Chinese herbal medicine) particles (5 g three times daily), furosemide (20 mg once daily), and spironolactone (20 mg once daily). Throughout his periods of infection in other hospitals, his doctors once treated him with intravenously administered levofloxacin (500 mg once daily)/ciprofloxacin (200 mg every 12 hours)/Tazocin (piperacillin-tazobactam; 4.5 g every 8 hours)/latamoxef (2 g twice daily)/ceftriaxone (2 g once daily).

**Fig. 1:**
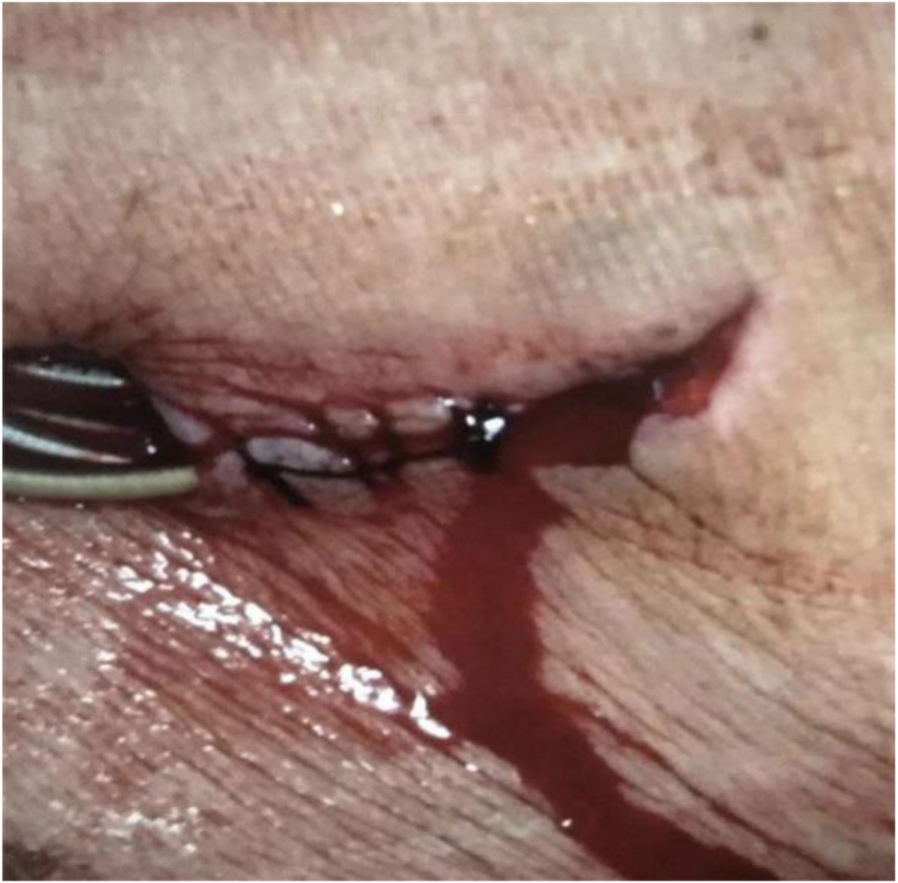
Appearance of the wound at the time of admission. Adherence of skin to the leads was eroded overtly and draining sinus can be observed on the right side of the upper chest

At the time of the new admission to our hospital, he was looking chronically ill. He was febrile with a temperature of 38.0 °C, and felt short of breath (New York Heart Association Functional Classification III). Oxygen saturation was 90–95% on room air. He was hemodynamically stable with a blood pressure of 165/74 mmHg, and heart rate of 63 beats per minute. He has smoked tobacco for more than 50 years and never drinks alcohol. A physical examination revealed adherence of skin to the device with overt erosion and draining sinus could be observed on the right side of his upper chest (Fig. 1). We could see pus when squeezing the surgical incision. A cardiovascular examination was unremarkable. No evidence of infective endocarditis was observed. A chest examination showed bilateral basal crepitations. Severe edema was found in his penis, scrotum, and lower extremities. Laboratory test values are summarized in Table 1. A transthoracic echocardiography (TTE) displayed that his left ventricular ejection fraction was 52%, and the result showed no evidence of vegetation attached to heart valves. Because of poor quality of TTE for diagnosis of infective endocarditis, a transesophageal echocardiography (TEE) was requested. However, our patient refused the examination firmly. Therefore, vegetation associated with device leads and endocardium could not be completely eliminated.

**Table 1:** Laboratory test results on admission

Test	Patient’s value	Normal range
WBC	8.10 × 10^9^/L	4–10
NEUT%	79.60%	50–70
PCT	0.67 ng/ml	0–0.1
CRP	14.05 mg/L	Inflammation >10.0
Urea	12.48 mmol/L	1.5–7.5
Creatinine	170 umol/L	32–106
e-GFR	30 mL/minute per 1.73 m^2^	>60
UA	539 umol/L	210–430
ALT	10 U/L	9–50
AST	22 U/L	15–40
Albumin	25 g/L	35–52
TCHOL	2.6 mmol/L	3.1–5.7
LDL_c	1.42 mmol/L	1.57–3.76
D-Dimer	2350 microg/L	80–500
Troponin I	<0.010 microg/L	0.01—0.023
CK	47 U/L	26–174
NT-proBNP	4150 ng/L	300–900

*ALT* alanine aminotransferase, *AST* aspartate aminotransferase, *CK* creatine kinase, *CRP* C-reactive protein, *e-GFR* estimated glomerular filtration rate, *LDL_c* low density lipoprotein cholesterol, *NEUT%* the percentage of neutrophil granulocyte, *NT-pro*BNP N-terminal pro-brain natriuretic peptide, *PCT* procalcitonin, *TCHOL* total cholesterol, *UA* uric acid, *WBC* white blood cell count

**Fig. 2:**
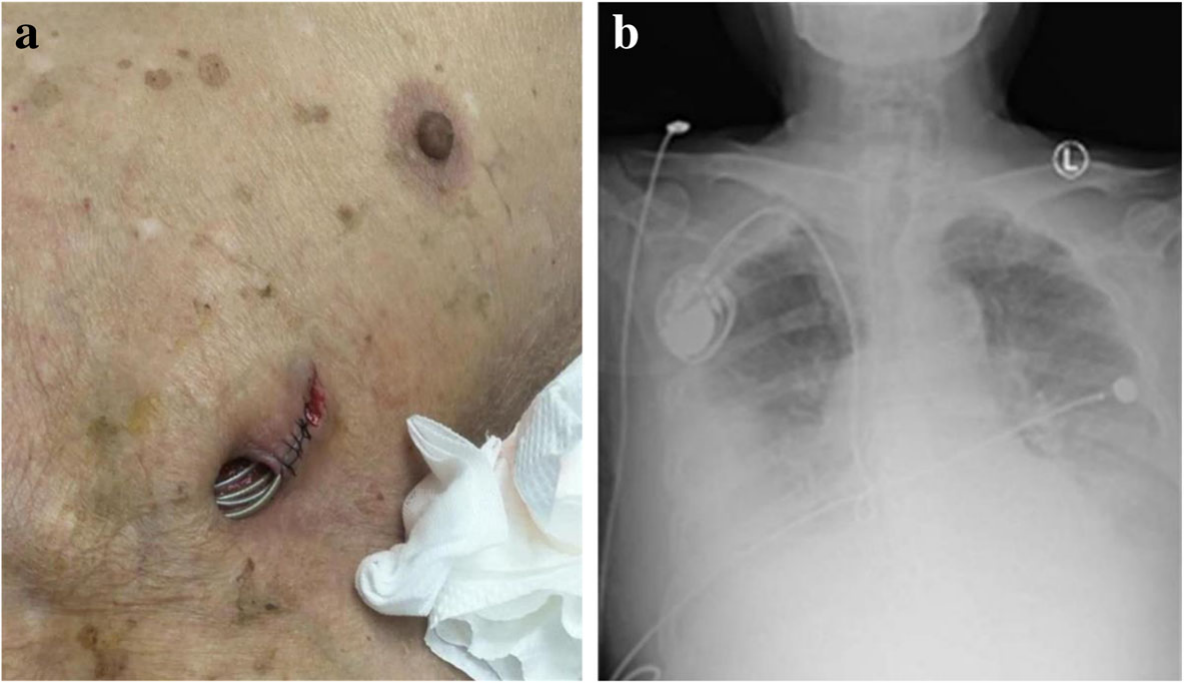
**a** Appearance of the wound after irrigation (second week in the hospital). **b** Preoperative chest X-ray showed the generator was in the infraclavicular region

Blood and pocket secretions of our patient were sampled and cultured for aerobic, anaerobic bacteria and fungi respectively. After this procedure, he started receiving an intravenously administered broad-spectrum antibiotic (moxifloxacin 400 mg once daily). Simultaneously, the wound was irrigated thoroughly with chlorhexidine, hydrogen peroxide, and saline every day (Fig. 2). On hospital day 7, the result of the blood culture revealed *Staphylococcus epidermidis*, and *Corynebacterium striatum* grew in the pus excretion culture. The pus Gram stain demonstrated Gram-positive, non-spore, rod-shaped bacteria, short but straight, which were arranged irregularly. No other strain was found in the Gram stain. The *S. epidermidis* was highly sensitive to penicillin, gentamicin, tetracycline, erythromycin, linezolid, vancomycin, and tigecycline; the *S. epidermidis* was moderately sensitive to levofloxacin, ciprofloxacin, and moxifloxacin, but not sensitive to cephalosporins. The *C. striatum* was highly sensitive to penicillin, cefoxitin, erythromycin, gentamicin, vancomycin, and teicoplanin; the *C. striatum* was moderately sensitive to clindamycin, but not sensitive to levofloxacin. Two sets of subsequent blood and secretion cultures after antibiotic therapy had confirmed the result. He was diagnosed as having complicated pacemaker pocket infection.

The intravenously administered antibiotic was changed to penicillin (3,200,000 IU every 8 hours) according to the antimicrobial drug susceptibility profile of our patient. Considering that he had severe infection, heart failure, and hypoalbuminemia, we treated him with intravenously administered immunoglobulin (2.5 g once daily), human albumin (10 g once daily), and furosemide (20 mg once daily). At the same time, fosinopril sodium (10 mg once a day), furosemide (20 mg twice daily), and spironolactone (20 mg twice daily) were taken orally to control hypertension and reduce severity of heart failure. Orally administered niaoduqing (Chinese herbal medicine) particles (5 g three times daily) were also taken to improve renal function. After 1 month of conservative treatment, he was afebrile and his heavy breathing had improved.

**Fig. 3:**
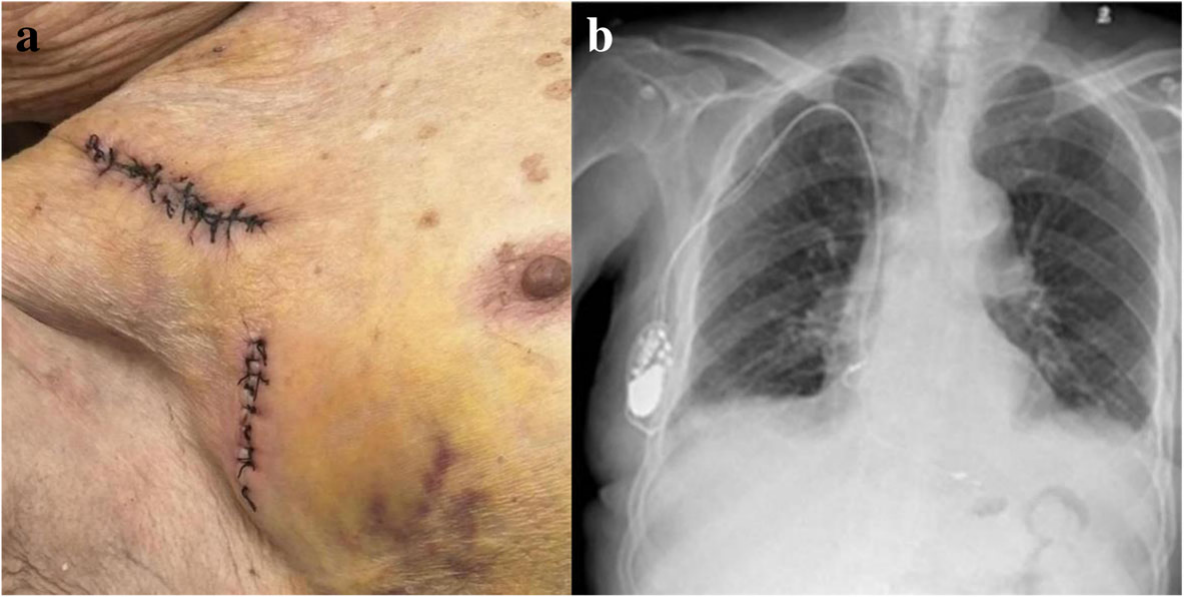
**a** Appearance of the wound after reimplantation. **b** Postoperative chest X-ray showed the generator was reimplanted in different position on the same side

The Holter monitor and pacemaker program suggested that he was not completely dependent on the pacemaker. Our preferred treatment strategy was for complete removal of both the generator and transvenous pacing leads, and we intended to implant an epicardial pacemaker in our patient if necessary. However, he rejected the treatment strategy, and refused to replace his generator because of economic factors. We had to attempt a novel device-preserving strategy considering our patient’s severe comorbidities. Subsequently, the generator was extracted and immersed in povidone-iodine for sterilization. The lead was disconnected from the generator, capped, and allowed to remain *in situ*. Because our patient was not completely dependent on the pacemaker, a temporary pacemaker was not used. His heart rate fluctuated from 33 to 68 times per minute after extracting pulse generator. Necrotic tissue was extensively debrided to shorten the time of wound healing. During this time, intravenously administered antibiotics and cardiac monitoring were continued. After 10 days, when blood culture was negative, the sterilized generator was successfully reimplanted in a different position on the same side (Fig. 3). The generator remained out of the pocket for 10 days in total. The surgical wound healed rapidly. Seven days after device reimplantation, he was discharged with an extensive list of medications: 10 mg of fosinopril sodium once daily; 20 mg of furosemide once daily; 20 mg of spironolactone once daily; 20 mg of trimetazidine dihydrochloride three times daily; 5 g of niaoduqing (Chinese herbal medicine) particles three times daily; and 150 mg of iron polysaccharide complex capsules once daily. No orally administered antibiotic was used after discharge.

**Fig. 4:**
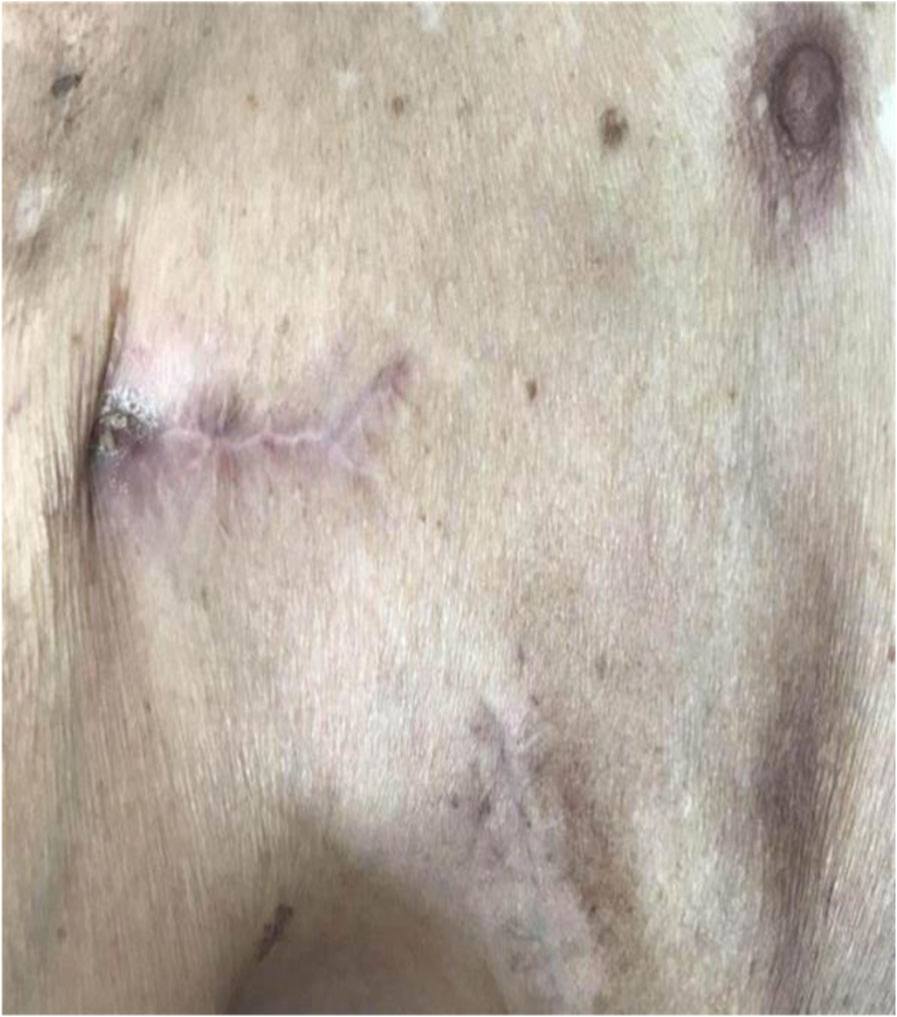
Complete healing of the wound (sixth month after discharge)

**Fig. 5:**
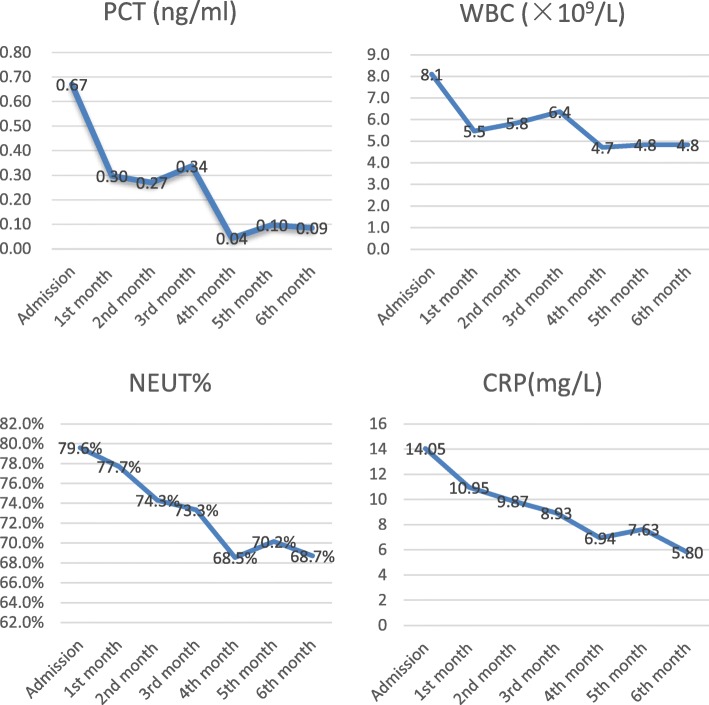
Clinical makers of infection were normal during a 6-month follow-up. *CRP* C-reactive protein, *NEUT* neutrophils, *PCT* procalcitonin, *WBC* white blood cells

**Fig. 6:**
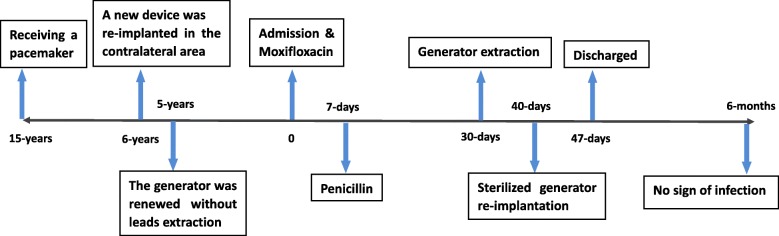
Timeline shows disease progression

He showed up to his follow-up appointments every month. There was no sign of wound dehiscence (Fig. 4) and the pacemaker worked properly during a 6-month follow-up. Clinical markers of infection were normal and recorded (Fig. 5). At the time of his last follow-up appointment, he was afebrile with a temperature of 37.2 °C. Oxygen saturation was 100% on room air. His blood pressure was 150/61 mmHg, and heart rate was 52 beats per minute. A physical examination revealed there were surgical scars but no sign of wound dehiscence on the right side of his upper chest. There was still mild edema in lower extremities, but he did not have obvious shortness of breath at rest. A chest examination showed no bilateral basal crepitations. A cardiovascular examination was unremarkable. No heart murmur could be heard. A neurological examination revealed that his functions of sensation and movement were normal, and he was able to carry out daily activities independently. A timeline to show disease progression is shown in Fig. 6.

## Discussion

We presented a patient with complicated generator pocket infection. Leads removal might not have been an option for him because he was at very high risk because of age and concomitant diseases. We treated him with partial generator removal and reimplantation of the sterilized pulse generator on the ipsilateral side. Few studies in the literature have reported such therapeutic strategy.

CIEDI is a serious cardiovascular disease and it is associated with a high mortality. In a large cohort of patients with CIEDI, the 30-day mortality rate was 5.5%, and 1-year mortality was 14.6% [8]. Erosion of any part of the CIED indicates contamination of the entire system, and complete device removal should be performed. Conservative antibiotic therapy combined with limited debridement and irrigation of infected sites without removal of the infected device system may lead to poor outcomes. Le *et al.* reported that antimicrobial therapy without device removal was associated with a sevenfold increase in 30-day mortality [8].

Although complete removal of an infected CIED is first-line therapy, there may be complications about device removal. According to previous statistics, the risk of major complications (for example, vascular laceration, death) approaches 2% for all extraction attempts [6]. Risk factors associated with procedural complications and death are not completely known. In the LExICon study [9], patients with a body mass index (BMI) <25 kg/m^2^ were more likely to experience major adverse events related to the lead extraction procedure. Some risk factors such as low BMI, renal disease, diabetes mellitus, and extraction for infection, increased the risk for death during their hospitalization. Another study confirmed the relevance of these risk factors. Brunner *et al.* produced a nomogram for the risk of 30-day all-cause death after leads extraction [10]. The factors with the highest predictive value for death were heart failure, older age, abnormal BMI, and extraction for infection [10].

The patient we described was elderly, and suffered from heart failure (New York Heart Association Functional Class III), chronic renal failure, and low BMI (18 kg/m^2^). The removal of the entire pacing system could predispose our patient to unexpected events. In addition, he preferred conservative therapy as he had experienced complete extraction of the pulse generator and leads on the contralateral side 6 years previously. As a result, we decided to partially remove the generator combined with conservative treatment (antibiotic therapy, debridement, and irrigation).

Some case reports and small case series suggested that salvage of an infected CIED may sometimes be successful. Lopez saved the pulse generators and leads of five patients by using mechanical means (scrubbing and pulsed lavage) and a closed antimicrobial irrigation system [11]. Tan *et al.* retrospectively analyzed 33 patients who initiated chronic antibiotic suppression without device removal, only 18% (6/33) developing into relapse within 1 year [12]. Peacock *et al*. reported on 127 patients for whom conservative management with device retention was attempted, 20% met the study definition for successful salvage [13]. Even with more serious forms of device infection, such as leads endocarditis, medical management without device removal may be successful. In the study of Tascini and colleagues [14], two out of nine patients with CIED endocarditis were too sick for the removal of their CIED, and were cured with 6 mg/kg of daptomycin without adverse event.

There have been some reports concerning the partial device removal of infected CIED [15–17]. Table 2 was used to compare our case with cases that were available in the literature. In those studies, incomplete removal resulted in greater rates of infection relapse. According to those studies, we may successfully manage the patient with conservative treatment and partial removal of device, but the likelihood of infection relapse in our patient was considerable. We should have a longer follow-up.

**Table 2:** Review of literature regarding salvage of cardiovascular implantable electronic devices

Study	Infection, *n*	Management, *n*	Complications, *n*	Success	Follow-up (months)
Present study, 2018	CIEDI (1)	Partial removal + conservative therapy + sterilized generator reimplantation (1)	0	100%	6
Lopez [11], 2013	CIEDI (5)	Scrubbing + pulsed lavage + closed antimicrobial irrigation system (5)	0	100%	19.2
Tan *et al*. [12], 2017	CIEDI (33)	Chronic antibiotic suppression (33)	Infection relapse (6)	82%	12
Peacock *et al*. [13], 2018	CIEDI (127)	Device retention + antibiotics (127)	Early failure of salvage (74); infection relapse (6); chronic suppression (7); death (14)	20%	6
Tascini *et al.* [14], 2012	CIED endocarditis (2)	Daptomycin (2)	0	100%	17
Margey *et al*. [15], 2010	CIEDI (13)	Partial removal or conservative therapy (13)	Infection relapse (8); death (1)	25%	36

*CIED* cardiovascular implantable electronic device, *CIEDI* cardiovascular implantable electronic device infection

It is sometimes difficult to determine the causative organism. This is because the results of each culture from different sites may suggest different organisms. Bongiorni *et al.* reported on one of the largest (1204 patients) microbiology studies in CIEDI [18]. They investigated 116 cases of materials from pockets and 359 cases where blood samples were obtained for culture. The results were consistent with those from electrodes in 59% and 35% of cases respectively. Golzio *et al*. gave a definition of causative organism as consistent species detected from at least two different sites [19]. Each blood culture was counted as a different material, and pocket material was considered a single site. Nine sets of blood and pocket excretion samples of our patient were cultured for aerobic, anaerobic bacteria and fungi. Three blood cultures revealed *S. epidermidis*, four revealed other coagulase-negative *Staphylococcus*, and the other two were negative. Four pocket excretion cultures revealed *C. striatum* and the other five were negative. Therefore, we can regard coagulase-negative *Staphylococcus* as the causative organism. According to the study findings of Fukunaga *et al.*, the causative organism of the CIEDI was mainly *Staphylococcus aureus* and coagulase-negative *Staphylococci* (for example, *S. epidermidis*), but *S. aureus* showed a higher concordance in leads and pocket cultures than coagulase-negative *Staphylococci* [20]. Gram-positive bacteria (excluding *Staphylococcus*), such as *Corynebacterium* species, showed relatively low concordance, which meant a benign coexisting organism [20]. In the case of our patient, blood cultures were coagulase-negative *Staphylococcus* but *C. striatum* grew in the pocket excretion cultures. This could be explained by the use of antibiotic therapy previously and preexisting surgical tissue debridement before his new admission. Eventually, we identified coagulase-negative *Staphylococcus* as the causative organism while *C. striatum* tended to coexist as a benign organism. As with most infections, an antimicrobial drug initially should cover common organisms broadly and antibiotic administration should begin after collection of blood and excretion cultures. Narrowing of the antimicrobial spectrum should be based on antimicrobial drug susceptibility. The duration of antibiotic treatment after removal of an infected device varied in different studies. In general, the more residual devices left in place, the longer the duration of treatment. We used antimicrobial therapy for 47 days in total and for 7 days after reimplantation in our patient, which was in accordance with the guidelines [4].

The pulse generator of our patient was removed for sterilization. For patients who are pacemaker-dependent, temporary pacing is required as a bridge to the reimplantation of a new permanent device [7]. However, it has been associated with higher mortality [21], and increased risk of infection [22]. We daringly did not use a temporary pacemaker in our patient, although his heart rate was sometimes only 33 times per minute. Customary treatment for lead infection would involve contralateral implantation of a new device. Given complete contralateral venous occlusion and our patient’s rejection of a new generator, the sterilized prior generator was reimplanted in a different position on the same side connecting old electrodes. No report has introduced the surgical technique to date.

## Conclusions

We would posit that partial removal of infected generators combined with conservative treatment may be a proper treatment of complicated generator pocket infection, especially for those who are susceptible to cardiac complications or lack the necessary financial resources. Although not a widely accepted practice, reimplantation of the sterilized pulse generator on the ipsilateral side may be an option if a patient rejects a new device and contralateral vascular condition is not really suitable. Opting for such treatment should be at the consideration of the primary care physician based on the condition of the patient, and the pulse generator and remaining leads must be completely sterilized to eradicate infection. On the basis of a limited number of patients, further studies are needed to better define the optimal subpopulation that would benefit from the approach.

## Abbreviations

BMI: Body mass index
CIED: Cardiovascular implantable electronic device
CIEDI: Cardiovascular implantable electronic device infection
*C. striatum*: *Corynebacterium striatum*
*S. aureus*: *Staphylococcus aureus*
*S. epidermidis*: *Staphylococcus epidermidis*
TTE: Transthoracic echocardiography
